# A Gyroscope-Pendulum-Coupled Multilayer Triboelectric Nanogenerator for Omnidirectional Low-Frequency Ocean Wave Energy Harvesting

**DOI:** 10.3390/mi17091010

**Published:** 2026-08-26

**Authors:** Songhang Li, Zhenlong Xu, Zheming Zhang, Yiwen Zhu, Xiaohan Xu, Chengping Deng, Xinting Ge

**Affiliations:** 1 School of Mechanical Engineering, Hangzhou Dianzi University, Hangzhou 310018, China; lsh23010832@hotmail.com (S.L.); mingzhizheshui@gmail.com (Z.Z.); 24010305@hdu.edu.cn (Y.Z.); xiaohan@xuxiaohan.work (X.X.); 17369587350@163.com (C.D.); 24010702@hdu.edu.cn (X.G.); 2 School of Mechanical and Manufacturing Engineering, University of New South Wales, Sydney, NSW 2052, Australia

**Keywords:** triboelectric nanogenerator, wave energy harvesting, omnidirectional response, gyroscope-pendulum coupling, multilayer structure

## Abstract

Low-frequency, irregular water waves with continuously changing propagation directions are difficult to harvest efficiently using conventional power generation devices. This work proposes a gyroscope-pendulum-coupled multilayer triboelectric nanogenerator (GP-TENG), in which a multi-axis gyroscope mechanism, an inertial pendulum, and a helical-structured power generation module are integrated inside a spherical floating body. The gyroscope joints enable the pendulum to respond to waves arriving from any horizontal direction, while the heave and tilting motions of the floating body jointly drive periodic contact and separation of the multilayer triboelectric materials. Motor-driven platform and water tank experiments were conducted to investigate the effects of the number of generating layers, excitation frequency, translational stroke, swing amplitude, and external resistance on the output performance. In the controlled translational tests, the maximum root-mean-square open-circuit voltage, short-circuit current, and transferred charge reached 98.6 V, 2.3 μA, and 242 nC, respectively, and a maximum output power of 16.3 μW was obtained at a load of 81 MΩ. In the water tank, the GP-TENG showed a stable response near 1.42 Hz, with maximum output power of 3.45 μW at a 60 MΩ load. The generator successfully charged the capacitor, lit up LEDs, and powered a commercial temperature and humidity sensor. These results indicate that the GP-TENG provides a compact and low-cost approach for omnidirectional low-frequency wave energy harvesting and a distributed power supply for low-power marine electronic devices.

## 1. Introduction

Ocean energy is a renewable and clean energy source that can complement mature solar and wind technologies. However, ocean energy conversion still faces many challenges because marine resources are spatially dispersed, operating conditions vary considerably, and conventional hydropower technologies cannot be directly applied to open waters. Among the various forms of ocean energy, surface wave energy has attracted extensive attention because of its abundant reserves and accessibility, but its characteristic frequency is generally below 2 Hz, while the wave amplitude and propagation direction continuously vary [1,2,3,4].

Conventional electromagnetic generators usually have low efficiency under low-speed excitation and often require additional transmission or frequency-up conversion mechanisms. Triboelectric nanogenerators (TENGs), based on the coupling of contact electrification and electrostatic induction, can directly convert low-frequency and irregular mechanical motion into high-voltage electrical signals. Their simple structure, light weight, broad material selection, and relatively low manufacturing cost make them promising for harvesting high-entropy mechanical energy from water waves [5,6,7,8].

For blue energy harvesting, various TENG structural strategies have been developed to address the low-frequency and intermittent characteristics of waves. To enhance low-frequency response, cylindrical swing and rolling-sphere structures increase the output frequency by extending the internal motion after a single trigger [9,10], while spring-assisted multilayer structures achieve frequency up-conversion through mechanical resonance and simultaneously enlarge the effective triboelectric area [11]. For energy buffering and conversion, pneumatic membrane arrays distribute wave-induced air pressure among multiple generating units to improve the utilization of intermittent inputs [12], whereas pendulum structures employ gravitational potential energy and inertial delay to realize frequency multiplication or prolong the response duration [13,14]. For hybrid generation, triboelectric–electromagnetic structures use electromagnetic units to compensate for the insufficient low-frequency output of TENGs and broaden the effective operating band [15,16]. Overall, these strategies have achieved clear improvements in output frequency, space utilization, structural robustness, and environmental adaptability [17,18,19,20,21,22]. However, resonance-dependent devices are generally restricted by their matching bandwidth, and many wave-driven TENGs are still designed primarily for a single or preferred direction of motion.

Therefore, after addressing low-frequency conversion, adapting to wave inputs with continuously varying propagation directions becomes a further core requirement in device design. Existing omnidirectional or arbitrary-directional harvesting schemes can be classified into four routes according to their mechanisms [23]. First, the rolling capability of spheres or the mobility of liquids is used to achieve passive omnidirectional adaptation, but the limited solid–solid contact area results in a relatively low output power density, while liquid motion also requires consideration of sealing and related issues [24,25,26]. Second, spatially distributed units provide geometrically symmetric coverage but increase the complexity of wiring and signal management [27,28,29]. Third, multi-degree-of-freedom mechanical decoupling redirects multidirectional excitation toward the generating unit, but the additional frame increases device volume [30,31]. Fourth, hybrid adaptive strategies combining biomimetic structures, pneumatic buffering, or circuit control improve omnidirectional adaptability and output stability, but the added system functions also introduce more complex components and control stages [32,33,34,35].

Overall, existing omnidirectional harvesting schemes often obtain directional adaptability at the expense of structural compactness or energy density. Among these routes, gyroscopic mechanisms offer the distinct advantage of converting excitation from arbitrary horizontal directions into controllable internal motion, whereas spiral multilayer generating units can substantially increase the effective triboelectric area within a limited volume [36]. Although these two functions have generally been implemented separately in previous studies, compact passive structures that integrate directional decoupling with high-area multilayer conversion remain insufficiently investigated. Such structures enable vertical heave and arbitrary horizontal tilt to drive the same generating module.

Based on the above considerations, this work proposes a floating gyroscope-pendulum-coupled multilayer triboelectric nanogenerator (GP-TENG) composed of a nested gyroscope mechanism, an inertial pendulum, an 11-layer power generation module, and a spherical wave-responsive shell. The vertical displacement of the floating body directly compresses and releases the multilayer generating units, while tilting motion from any horizontal direction is converted by the pendulum into contact-separation motion between triboelectric interfaces. The device performance was systematically evaluated through structural and mechanical analyses, controlled translational and swing tests, and water tank experiments. The results verify the feasibility of harvesting omnidirectional low-frequency wave energy using a simple mechanical configuration at low cost.

## 2. Results and Discussion

### 2.1. Structure of the GP-TENG

**Figure 1:**
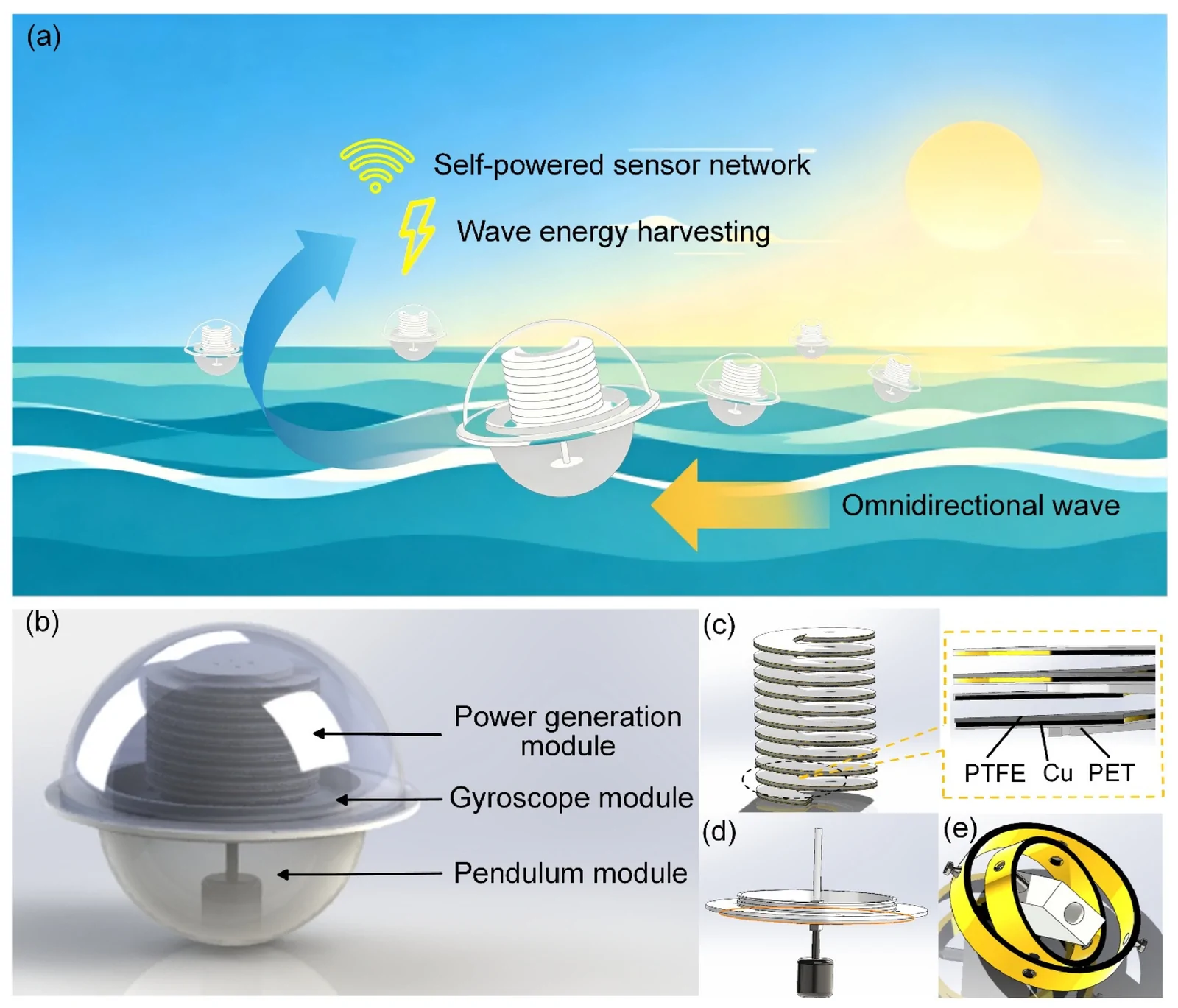
Application concept and structural design of the GP-TENG. (**a**) Schematic of floating GP-TENGs for omnidirectional wave energy harvesting and self-powered marine sensor networks; (**b**) Overall GP-TENG structure, including the power generation module, gyroscope module, and pendulum module; (**c**) Helical-structured power generation module and triboelectric layer configuration; (**d**) Pendulum module; (**e**) Nested gyroscope module.

Figure 1a presents the application concepts of distributed GP-TENGs as floating wave energy harvesting nodes for self-powered marine sensor networks. As shown in Figure 1b, the GP-TENG consists of a power generation module, a gyroscope module, and a pendulum module. This arrangement allows the multiple triboelectric layers to be driven by both heave and tilting motions, without requiring separate transduction units for different wave motion components.

As shown in Figure 1c, the power generation module contains 11 contact-separation triboelectric layers. A parallel spiral framework formed by commercially available spring-like polymer rings supports annular PET films, which act as substrates. Cu foil and polytetrafluoroethylene (PTFE) film were selected as the triboelectric materials due to their substantial difference in electronegativity [30]. Cu foil serves both as the positive triboelectric material and as the electrode, while PTFE covers the opposing Cu surface and acts as the negative triboelectric material. The helical multilayer structure provides high area-to-volume ratio, thereby generating more triboelectric charges per unit volume.

The pendulum module shown in Figure 1d consists of upper and lower rods and a threaded connector at the center. The gyroscope module in Figure 1e includes two concentric Cu rings, bearings, and screws. The diameters of the two rings are 50 mm and 40 mm, respectively, and their widths are 10 mm. The orthogonal rotation axes of the two rings allow the pendulum to rotate with multiple degrees of freedom via the connector. Therefore, when the floating shell tilts in any horizontal direction, the pendulum tends to retain its original orientation because of inertia, producing relative motion between the rods and the shell. The lower end of the helical framework was fixed to the central support plate, while the upper end was connected to the upper rod.

The outer diameters of the upper and lower acrylic hemispherical shells are approximately 230 mm and 180 mm, respectively, and both have a wall thickness of 2 mm. The overall dimensions of the assembled GP-TENG are approximately 250 mm (diameter) × 265 mm (height), and the measured mass of the assembled prototype is approximately 1.6 kg. The central support plate and the upper and lower end plates of the power generation module were fabricated by 3D printing with nylon. A rubber sealing ring was installed at the shell-connection interface, and a stainless-steel cylindrical counterweight, 50 mm in diameter and 50 mm in height, was mounted at the bottom of the lower shell to enhance self-restoring capability. The transparent shell also allows direct observation of the internal mechanism during experiments.

### 2.2. Working Principle

The GP-TENG is driven through two coupled motion paths. During heave motion, inertia causes the vertical movement of the helical layers to lag behind that of the shell, directly producing periodic compression and release. During roll or pitch motion, the inertial pendulum rotates relative to the shell through the gyroscope joints. This motion drives the upper rod to pull the top end plate, inducing the separation and contact of the helical layers. Since the gyroscope module provides rotational degrees of freedom around different horizontal axes, omnidirectional waves can excite the coupled motion between the pendulum and the power generation module. The repeated deformation of the helical structure under combined wave excitation can also be directly observed in Supplementary Video S1.

**Figure 2:**
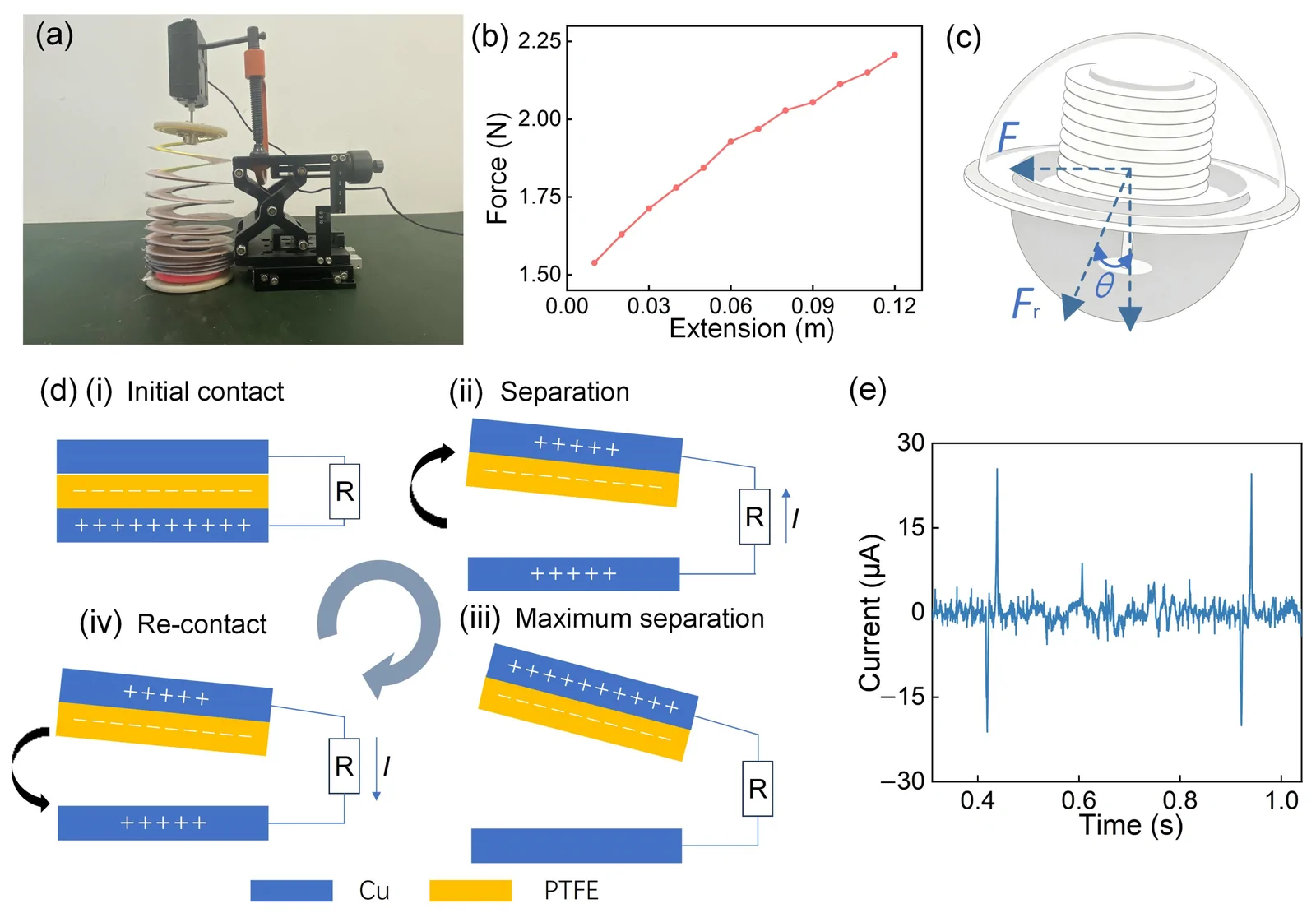
Mechanical and electrical working principles of the GP-TENG. (**a**) Restoring force measurement setup; (**b**) Restoring force—extension relationship; (**c**) Force analysis of the pendulum module; (**d**) Power generation process of the triboelectric layers; (**e**) Short-circuit current signal of the 11-layer GP-TENG under translational excitation at 2 Hz.

The mechanical response of the power generation module is closely related to the natural frequency of the pendulum. The helical structure introduces additional stiffness and mass into the pendulum system. To characterize its equivalent stiffness, the force–displacement response was measured using the push–pull force gauge and lifting setup shown in Figure 2a. The measured force *F*_m_ increased monotonically with extension *x* and was fitted linearly as follows:

(1) $$F_{m}\;=1.5314\;+\;5.8736x$$

The intercept of 1.5314 N represents the initial load at zero extension, including the gravitational and assembly-preload contributions of the module, whereas the fitted slope corresponds to an equivalent stiffness of 5.8736 N·m^−1^. Therefore, the deformation-induced restoring force *F*_r_ is expressed as

(2) $$F_{r}=5.8736x$$

Figure 2c presents the simplified force analysis. When the pendulum is displaced by an angle *θ*, the component of the restoring force opposing the pendulum motion is given by

(3) $$F=F_{r}\sin\theta$$

where *F* is the effective restoring force component acting against the pendulum motion. The rotational equation of motion of the pendulum is established based on the moment balance about the rotation center. During pendulum motion, the main restoring moments are the gravitational restoring moment and the additional restoring moment generated by deformation of the helical structure. Therefore,

(4) $$ml^{2}\frac{d^{2}\theta }{dt^{2}}=-Fl-mgl\sin\theta$$

The nonlinear equation of motion was written as

(5) $$\frac{d^{2}\theta }{dt^{2}}+\frac{F}{ml}+\frac{g}{l}\sin\theta =0$$

where *m* is the equivalent lumped mass associated with the pendulum motion, and *l* is the effective upper-rod length. In the simplified model adopted in this work, the velocity-dependent damping effects caused by bearing friction, air resistance, and internal material dissipation are considered small relative to the restoring effects and are therefore neglected.

The equation was solved using the Runge–Kutta method, yielding a vibration frequency of 1.51 Hz. This frequency lies within the target low-frequency range of 1–2 Hz and is close to the optimal response frequency observed experimentally.

Power generation of the GP-TENG follows the vertical contact-separation mechanism shown in Figure 2d. Stage i is the beginning of the cycle, when the PTFE film carries a negative charge, the lower Cu electrode carries a positive charge, and no current flows through the load. When the two materials begin to separate under an external force, driven by the potential difference, electrons pass through the load to the lower Cu electrode and produce instantaneous current (stage ii). When separation is large enough, all positive charges are concentrated on the upper Cu electrode, the potential difference disappears, and no charge passes through the load (stage iii). When the two layers approach each other again under the external force, the potential difference causes the electrons to move back toward the upper Cu electrode, producing instantaneous current in the opposite direction through the load (stage iv). Repetition of this cycle produces alternative current output. Figure 2e presents the short-circuit (SC) current signal of the 11-layer GP-TENG under translational excitation at 2 Hz. The alternating current pulses with opposite polarities indicate electron transfer in opposite directions during the separation and re-contact processes, which is consistent with the contact–separation mechanism illustrated in Figure 2d.

### 2.3. Output Performance of the GP-TENG

**Figure 3:**
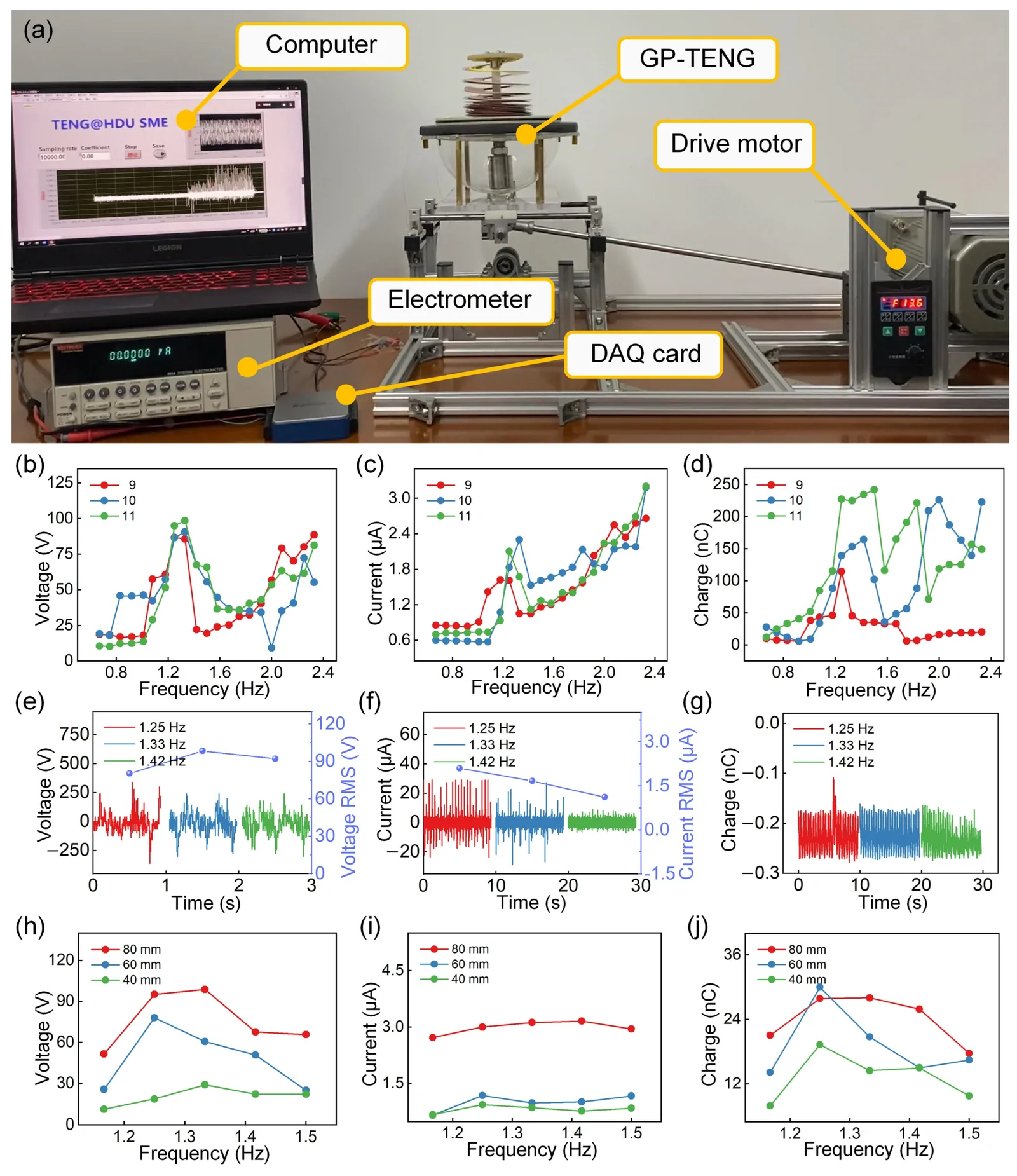
Output performance of the GP-TENG on the translational platform. (**a**) Motor-driven translational platform; (**b**–**d**) Effects of the number of triboelectric layers on voltage, current, and transferred charge; (**e**–**g**) Output voltage, current, and charge responses at 1.25, 1.33, and 1.42 Hz; (**h**–**j**) Effects of translational stroke on the electrical outputs.

To evaluate the output performance of the GP-TENG, a motor-driven slider-crank platform was used to provide repeatable translational excitation, as shown in Figure 3a. The open-circuit (OC) voltage was measured using a digital oscilloscope (MDO-2204EC, GW Instek, New Taipei City, Taiwan, China), while the short-circuit current and transferred charge were measured using an electrometer (Model 6514, Keithley Instruments, Solon, OH, USA). The electrical signals were synchronously acquired using a data acquisition card (NI-9239, National Instruments, Austin, TX, USA) and recorded through a LabVIEW-based data acquisition system.

To balance compactness and high output, the number of triboelectric layers was increased from 9 to 11 to investigate its impact. As shown in Figure 3b–d, all three configurations exhibit the first distinct response peak within 1.2–1.3 Hz. The 11-layer device produces the highest OC voltage of 98.6 V at 1.33 Hz and the maximum transferred charge of 242 nC at 1.50 Hz, while the maximum SC current (2.3 μA) occurs in the 10-layer configuration. Based on the combined voltage, current, and transferred charge results, the 11-layer configuration exhibited the highest overall electrical output and was therefore selected as the power generation module for all subsequent experiments.

A preliminary frequency sweep identified the main favorable translational response range as approximately 1.2–1.4 Hz; therefore, 1.25, 1.33, and 1.42 Hz were selected for representative waveform comparison. Figure 3e–g compare electrical outputs at 1.25, 1.33, and 1.42 Hz. The voltage response was strongest near 1.33 Hz, while the amplitudes and waveform regularity of the current and charge signals varied with frequency. Observations of the dynamic behaviors reveal that under high-frequency conditions, severe lateral flapping occurs between adjacent spiral layers, introducing additional sliding friction. This prolongs the charge transfer time and reduces the transfer efficiency, ultimately leading to a decay in the output current.

The influence of translational stroke was investigated using crank lengths of 40, 60, and 80 mm. Except for a few charge data points, the voltage, current, and transferred charge all go up with increasing stroke (Figure 3h–j). This trend is mainly attributed to the greater contact-separation distance and velocity resulting from the more deformation of the helical structure.

**Figure 4:**
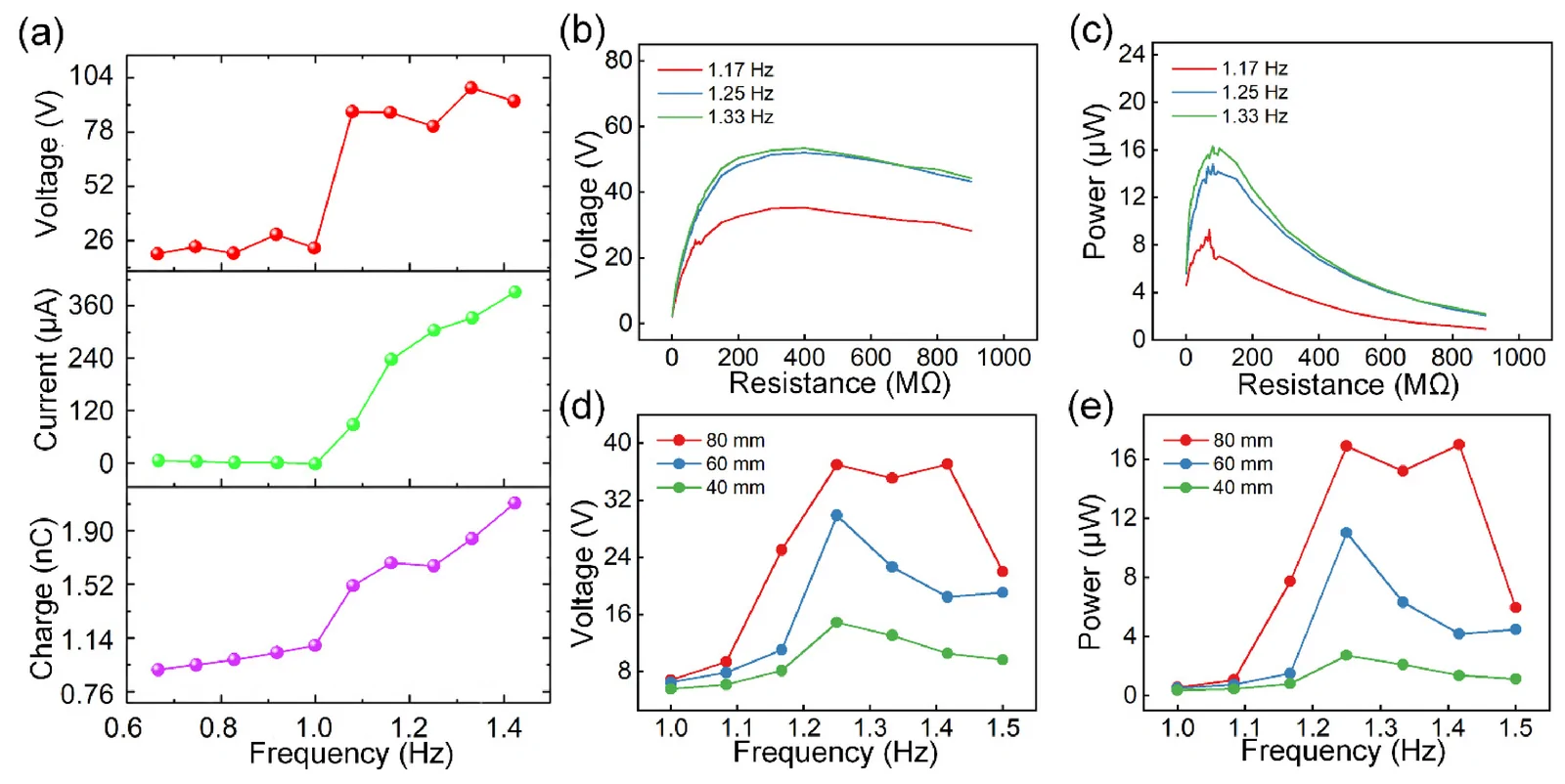
Swing performance and impedance matching experiments of the GP-TENG. (**a**) Electrical outputs under swing excitation; (**b**,**c**) Output voltage and power across external resistances at 1.17, 1.25, and 1.33 Hz; (**d**,**e**) Output voltage and power across the matched load for different translational strokes.

The electrical performance of the gyroscope-pendulum-coupled mechanism was further evaluated under swing excitation. A 40 mm crank was used for the swing tests, and the resulting swing amplitude was 32°. As shown in Figure 4a, the output increased markedly with excitation frequency. The measured maximum root-mean-square (RMS) OC voltage, SC current, and transferred charge are 98.4 V, 2.07 μA, and 392 nC, respectively. These values are comparable to or higher than those obtained under translational excitation with the stroke of 80 mm, indicating that the pendulum effectively converted tilting motion of the shell into deformation of the triboelectric layers.

The output characteristics were investigated by connecting external resistances across the device. A representative favorable operating point of approximately 1.25 Hz was identified in the translational tests; therefore, the adjacent frequencies of 1.17, 1.25, and 1.33 Hz were selected for impedance matching. Figure 4b,c show the output voltage and power across the external resistances at translational frequencies of 1.17, 1.25, and 1.33 Hz. As the load resistance increases, the load voltage gradually rises and approaches saturation, whereas the output power exhibits a clear peak. With the matched resistance of 81 MΩ, the output powers at 1.17, 1.25, and 1.33 Hz were 7.7, 14.8, and 16.3 μW, respectively. The results in Figure 4d,e again demonstrate that a larger translational displacement produces higher voltage and power. At a translational frequency of 1.42 Hz and a crank length of 80 mm, the maximum output power reached 17 μW.

### 2.4. Device Applications

**Figure 5:**
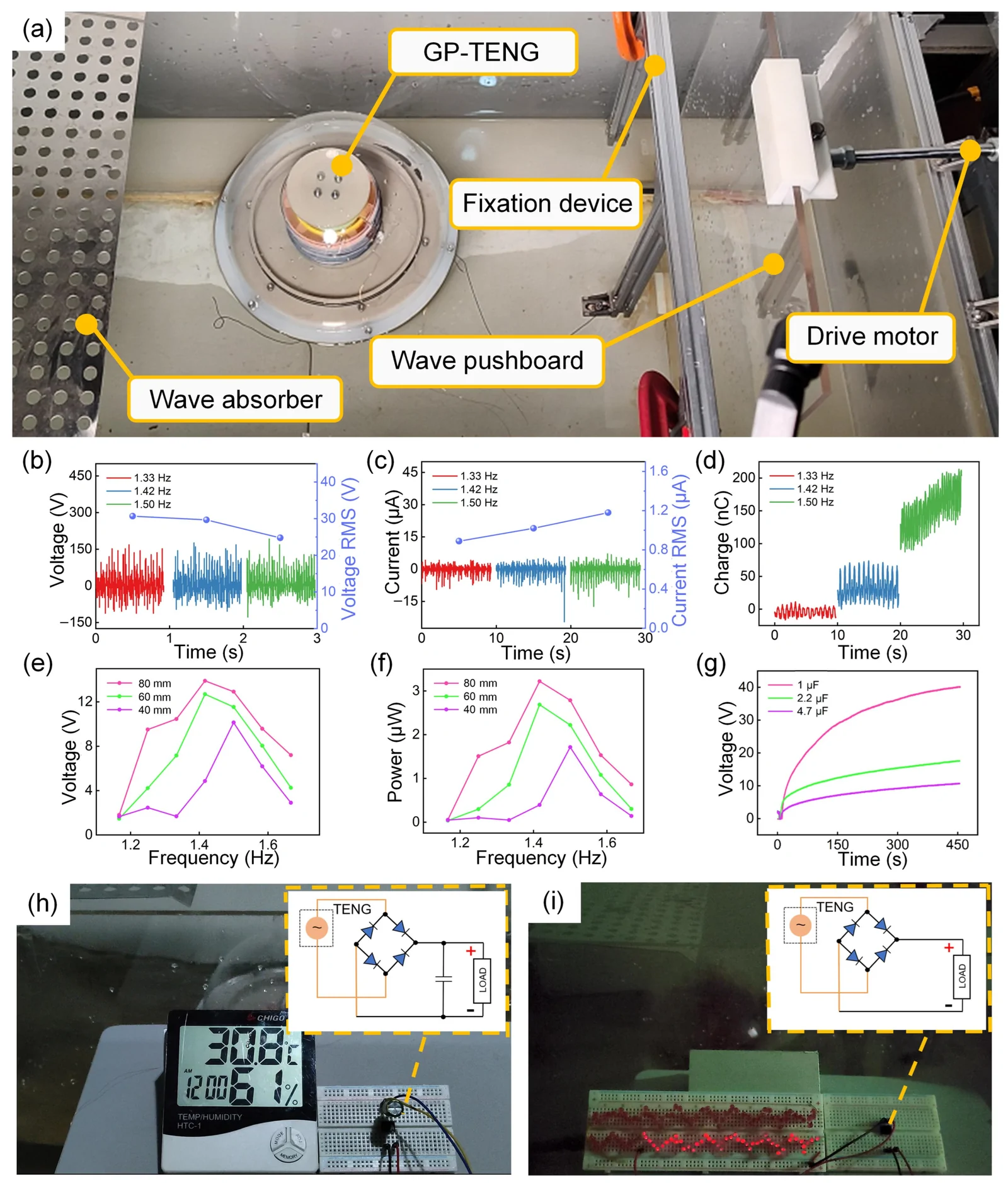
Water tank experiments and application demonstrations. (**a**) Water tank experimental system, including the GP-TENG, wave pushboard, drive motor, and wave absorber; (**b**–**d**) Voltage, current, and transferred charge at 1.33, 1.42, and 1.50 Hz; (**e**,**f**) Outputs at different crank lengths; (**g**) Charging curves of 1, 2.2, and 4.7 μF capacitors; (**h**) Demonstration of the GP-TENG powering a commercial temperature and humidity sensor; and (**i**) Demonstration of the GP-TENG lighting up 49 LEDs connected in series.

To evaluate the dynamic responses of the generator under actual water waves, the water tank experimental system was constructed, as shown in Figure 5a. A motor-driven acrylic wave pushboard generated periodic waves, and a wave absorber was used to reduce reflected waves.

The water-tank output curves exhibited a pronounced single peak mainly within 1.33–1.50 Hz, close to the theoretical natural frequency of 1.51 Hz; therefore, 1.33, 1.42, and 1.50 Hz were selected for waveform comparison. For the wave frequencies of 1.33, 1.42, and 1.50 Hz, the maximum OC voltage, SC current, and transferred charge measured were 30.7 V, 1.2 μA, and 12 nC, respectively. According to the waveforms, the RMS voltage decreases, whereas the other two outputs increase with frequency. Significant variations are observed in the charge waveforms. While the waveforms remain highly regular at 1.33 Hz and 1.42 Hz, it exhibits a fluctuating upward trend at 1.5 Hz. This is probably due to the flapping of the generating layers at this frequency, which subsequently causes sliding friction. Therefore, 1.42 Hz is selected as the optimal frequency due to the excellent trade-off between output amplitude and waveform stability (Figure 5b–d).

As displayed in Figure 5e,f, when connected to the matched resistance of 60 MΩ in the water tank, the output voltage and power generally rise with increasing crank lengths. However, the values are smaller than those measured in the motor-driven platform. This discrepancy is primarily attributed to the substantial hydrodynamic losses (e.g., turbulence and viscous dissipation) during the mechanical-to-hydraulic energy conversion, leading to less electrical energy generated.

The GP-TENG was then used to charge the capacitors. The AC output of the GP-TENG was first converted into DC using a DB107 bridge rectifier, which served as the rectification circuit, and then used to charge the corresponding capacitor. Figure 5g compares the charging curves of 1, 2.2, and 4.7 μF capacitors at a wave frequency of 1.42 Hz and a crank length of 40 mm. The 1 μF capacitor was charged to above 10 V within 60 s, the 2.2 μF capacitor required approximately 90 s, and the 4.7 μF capacitor approached 10 V after more than 400 s. The GP-TENG was further used to power electronic devices. As shown in Supplementary Video S2, to power a commercial temperature and humidity sensor, a 1000 μF capacitor was first charged by the GP-TENG through the DB107 rectification circuit for approximately 3 min and was then connected in parallel with the sensor, successfully activating its display (Figure 5h). As shown in Supplementary Video S3, the GP-TENG could also directly light up 49 LEDs connected in series through the same DB107 rectification circuit (Figure 5i). These demonstrations indicate that the GP-TENG, when combined with rectification and energy storage circuits, can supply power to electronic devices.

## 3. Conclusions

This work developed a gyroscope-pendulum-coupled multilayer triboelectric nanogenerator (GP-TENG) for harvesting omnidirectional low-frequency ocean wave energy. The nested gyroscope mechanism allows the inertial pendulum to respond to waves from different horizontal directions, while the helical-structured power generation module converts vertical heave and angular tilting motions into repeated contact-separation motions. The theoretical natural frequency of the device was 1.51 Hz. Under controlled translational excitation, the GP-TENG output 98.6 V, 2.3 μA, and 242 nC, and a maximum power of 16.3 μW was obtained across the matched resistance of 81 MΩ. In the water tank, the device operated stably near 1.42 Hz, with maximum outputs of 30.7 V, 1.2 μA, and 12 nC, and a peak power of 3.45 μW with a 60 MΩ load. After rectification, the device successfully charged capacitors, illuminated 49 LEDs in series, and powered a commercial temperature and humidity sensor. In a word, this work provides a compact and low-cost route for distributed power supply to low-power marine sensing nodes.

## Data Availability

The data that support the findings of this study are available from the corresponding author upon reasonable request.

## Supplementary Materials

The following supporting information can be downloaded at: https://www.mdpi.com/article/10.3390/mi17091010/s1, Video S1: Water tank experiments, Video S2: Temperature and humidity sensor powered by the GP-TENG, Video S3: 49 LEDs lighted up by the GP-TENG.
